# Everybody's Column

**Published:** 1892-03-05

**Authors:** 


### EVERYBODY'S COLUMN.

A STRONG tincture of aconite, mixed in the proportion of five or six drops in half a tumbler of water, and administered in doses of a teaspoonful at frequent intervals, is said to be a certain antidote to the poisonous sting of the scorpion. To be efficacious, it must like most remedies, be taken in time.

As several cases had been reported from time to time in America of poisoning by eating wild parsnip, a physician set to work to investigate the cases, and found that it was not wild parsnip but water hemlock which had given rise to poisonous symptoms. This is only one of many cases of inaccurate descriptions which are so misleading.

SIMPLE remedies are often the best. A writer in a German periodical advocates the use of ordinary soap as a corrective of the irritation arising from mosquito bites. As he lives in Africa he writes authoritatively; he states that on all expeditions he carries a small piece of soap for this purpose with him, and that if he is bitten he at once rubs a lather over the affected part, whereupon the burning is promptly relieved, and the pain soon ceases.

ODYSSEUS is often described by Homer as a man "of many devices," and amongst other devices he was apparently thoroughly acquainted with the best means of disinfection. In the twenty-second book of the Odyssey after the slaughter of the wooers Odysseus says to Eurycleia "Bring sulphur, old nurse, that cleanses all pollution and bring me fire that I may purify the house with sulphur." This might be some modern doctor demanding the means of disinfecting some patient's room after a scarlet-fever case.

DR. LANGDON, of Cincinnati, has found benzine a valuable solvent agent in the preparation of the skin for antiseptic operations, which can penetrate and disinfect the pores of the parts to be operated upon in a manner which cannot be accomplished by any of the watery solutions in common use.

LONDONERS may hear with questionable satisfaction that the Thames is not the only river which is a source of suspicion and alarm to those who live on its banks. The Croton has become a bye-word in New York, and the Hudson is said to supply to the inhabitants of Albany an infinite amount of food for the propagation of fever. Civilisation, undoubtedly, has its drawbacks, and there are many who would prefer the arcadian days when a river was a source of inspiration to the poet instead of being the subject of the anathemas of the sanitarian.

NOT so long ago the Meteorological Office astonished an incredulous people by assuring them that they had wasted a good deal of verbal energy over the unusual amount of the rainfall of the past year; for it was the time when the rain fell, and not its amount, which might excusably give rise to warmth of expression. It seems to be one of the tasks of this office to disabuse the public of false notions which it too fondly holds. We are now told authoritatively that the majority of lightning conductors in this country, instead of being a protection, as they are meant to be, are a source of great danger, owing to the imperfect and unscientific way in which they have been fixed. This will be cheerful news to a good many people.

